# 1,25(OH)_2_D_3_ increase osteogenic potential of human periodontal ligament cells with low osteoblast potential*

**DOI:** 10.1590/1678-7757-2024-0160

**Published:** 2024-11-22

**Authors:** Bruno Cazotti PEREIRA, Catharina Marques SACRAMENTO, Enilson Antonio SALLUM, Mabelle de Freitas MONTEIRO, Renato Corrêa Viana CASARIN, Marcio Zaffalon CASATI, Karina Gonzales SILVÉRIO

**Affiliations:** 1 Universidade Estadual de Campinas Faculdade de Odontologia de Piracicaba Piracicaba SP Brasil Universidade Estadual de Campinas – UNICAMP, Faculdade de Odontologia de Piracicaba, Piracicaba, SP, Brasil.

**Keywords:** Calcitriol, Osteoblast, Cementoblast, Periodontal Dental Ligament Mesenchymal Progenitor Cells, Differentiation, Asporin, Bone Morphogenetic Protein 2

## Abstract

Periodontal dental ligament mesenchymal stem cells (PDLMSCs) play a major role in periodontal tissue regeneration by the neoformation of root cementum and alveolar bone. These cells are highly heterogeneous, and many present low potential to renovate the hard tissue damaged by periodontal disease. A previous study found that the low osteoblast/cementoblast (O/C) differentiation potential of PDLMSCs is related to high asporin (*ASPN*) expression, which was identified as a negative regulator of PDL cells differentiation and mineralization, suppressing *BMP-2*-induced O/C differentiation.

**Objective:**

This study aimed to investigate whether 1,25(OH)_2_D_3_ treatment could stimulate the O/C differentiation of periodontal ligament mesenchymal progenitor cells characterized as low osteoblast potential (LOP), by asporin and bone morphogenetic protein-2 alteration.

**Methodology:**

Three LOP cell populations were cultured in standard medium (CONTROL), osteogenic medium (OM), and osteogenic medium associated with 1 nM of 1,25(OH)_2_D_3_ (OM + VD). The following assays were performed: 1) MTT to evaluate metabolic activity; 2) gene expression for asporin (*ASPN*), bone morphogenetic protein-2 (*BMP-2*), runt-related transcription factor 2 (*RUNX2*), alkaline phosphatase (*ALP*), osteocalcin (*OCN*), and vitamin D receptor (*VDR*) using qRT-PCR; 3) *BMP-2* extracellular expression; and 4) quantification of mineralized nodule deposition by Alizarin Red Staining. Data were subjected to two-way ANOVA and Tukey’s test (P<0.05).

**Results:**

The results showed that the 1,25(OH)_2_D_3_ treatment did not affect the cell viability, as demonstrated by metabolic activity increase over the 10 days in culture. After 14 days of 1,25(OH)_2_D_3_ treatment, the mRNA levels for *ASPN* and *VDR* decreased (P<0.05), while *BMP-2* transcripts and extracellular expression increased (P<0.05). In parallel, *RUNX2, ALP*, and *OCN* gene expression was upregulated by 1,25(OH)_2_D_3_ treatment, resulting in an increase of mineral nodule deposition *in vitro* (P<0.05).

**Conclusions:**

These data show that 1,25(OH)_2_D_3_ improves osteoblast/cementoblast differentiation of low osteoblast potential accompanied by alterations in *ASPN* and *BMP-2* expression.

## Graphical abstract

**Figure:**
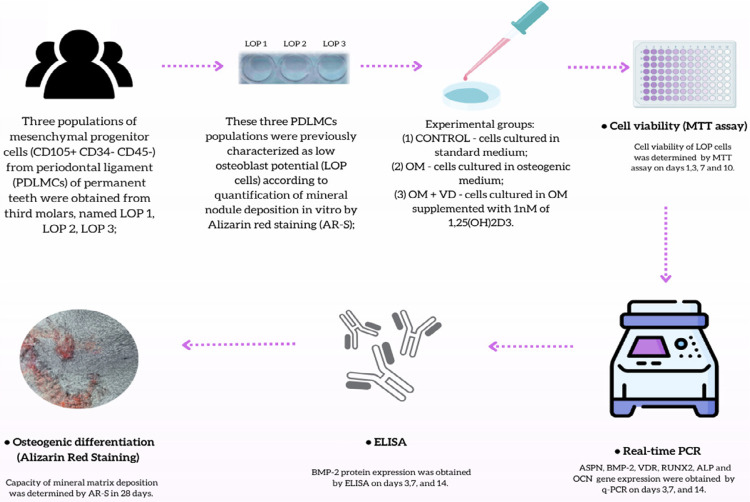
* This article is derived from a master’s dissertation

## Introduction

One of the biggest challenges of periodontal therapy has been the regeneration of lost periodontal ligament, cementum, and alveolar bone during the process of inflammatory periodontal disease. Numerous clinical therapies have been proposed to regenerate the periodontium, such as guided tissue regeneration, root conditioning, enamel matrix derivative protein, bone graft, and growth factors.^1-3^ However, periodontal regeneration has not been achieved completely and predictably with current techniques.^4,5^ That may occur due to periodontium being a complex structure comprised of soft and hard tissues, and the regeneration process requiring new cementum formation on previously exposed root surfaces, the synthesis of fibers, and the insertion into newly formed cementum and bone.^6,7^

Studies have shown that the neoformation of alveolar bone and mainly of root cementum depends on the migration and proliferation of remnant mesenchymal progenitor cells from periodontal ligament (PDLMCs) into the periodontal defect, followed by the differentiation into cementoblast or osteoblast cells (O/C).^8,9^ Nevertheless, PDL is constituted by heterogeneous cell populations, and the great amount of mesenchymal progenitor cells presents low potential to renovate the hard tissue damaged by periodontal disease.^10-14^ In previous studies performed by our research group, we could observe that even when cells were purified for CD105/endoglin/SH2 antigen (PDL-CD105^+^ cells), a specific mesenchymal stem cell marker, PDLMCs retained their heterogeneity and only few of them showed ability to produce mineralized matrix *in vitro*.^14-16^ Furthermore, it was found that PDL-CD105^+^ cells committed to O/C phenotype had notable variation in relation to mineralized matrix deposition *in vitro*.

Under osteogenic condition, PDL-CD105^+^ characterized as low osteoblast potential (LOP) cells showed a downregulation of the gene expression for osteoblast markers such as, runt-related transcription factor 2 (*RUNX2*), alkaline phosphatase *(ALP),* osteocalcin (*OCN*), and, for bone morphogenetic protein-2 (*BMP-2*),^16^ which is a potent inductor of O/C differentiation.^17^ LOP cells express high asporin (*ASPN*)/periodontal ligament protein-1 (*PLAP-1*) mRNA levels, which was identified as a negative regulator of PDL cells differentiation and mineralization suppressing *BMP-2*-induced osteoblast differentiation.^18^

Bone morphogenetic proteins (BMPs) have been strongly associated with osteogenic differentiation of mesenchymal stem cells (MSCs).^19^ Studies have confirmed that *BMP-2* accelerates the osteoblast differentiation of PDLMCs and enhances the expression of proteins associated with osteogenic differentiation, such as *ALP*, and *OCN*, thus promoting bone formation.^20-22^ In this way, the regulation of signaling pathways associated with osteogenesis may be a viable approach to modulate gene expression and change the phenotype of LOP cells, improving the predictability of periodontal tissue regeneration.

Vitamin D is a crucial fat-soluble vitamin that plays a fundamental role in various physiological processes within the human body.^23^ Vitamin D_3_ is metabolized by hydroxylation in two steps: the first hydroxylation occurs in the liver to form 25-hydroxyvitamin D3 (25OHD_3_), the main circulating metabolite of vitamin D_3;_^23^ the second one occurs in the kidney to form 1α,25(OH)_2_D_3_ (1,25D) the biologically active metabolite of vitamin D.^23^ The vitamin D receptor (*VDR*) is a nuclear receptor found in abundance in the key organs involved in calcium balance, including the periodontal ligament, intestine, bones, kidneys, and parathyroid glands.^24-26^ Recognized for its role in calcium homeostasis and bone health, emerging research has highlighted Vitamin D significance in maintaining periodontal health,^27^ since it also plays an important role in immunity, in the cardiovascular system, as well as in diabetes, cancer, and chronic illness.^28^ In addition, vitamin D could enhance the osteogenic differentiation of PDLMCs under inflammatory conditions by inhibiting *ASPN* expression transcriptionally.^29^

In this context, the study aimed to understand the effect of 1,25(OH)_2_D_3_ on periodontal ligament cells, more specifically to evaluate if vitamin D_3_ could alter the expression of *ASPN*, favoring *BMP-2* mediated LOP cell differentiation towards osteoblast/cementoblast phenotype. In this sense, we sought to determine the possible alterations and modulations to osteogenic differentiation that 1,25(OH)_2_D_3_ provided for three LOP cells. The null hypothesis is that there is no significant difference between OM and OM + VD groups in improving osteoblast/cementoblast potential of LOP cells.

## Methodology

### Cell culture

This study was approved by the Institutional Review Board of Piracicaba School of Dentistry of Campinas State University (CAAE 63957722.0.0000.5418), São Paulo, Brazil. Three populations of PDLMCs cells were exposed to 1,25(OH)_2_D_3_ and assessed for cell viability, *ASPN, BMP-2, VDR, RUNX2, ALP*, and *OCN* gene expression, in addition to *BMP-2* protein expression levels and osteogenic differentiation potential.

The three populations of mesenchymal progenitor cells (CD105^+^ CD34- CD45-) from periodontal ligament (PDLMCs) of permanent teeth were obtained from third molars, named LOP 1, LOP 2, LOP 3, and characterized in a previous study.^12^ Briefly, CD105^+^ enriched cell subsets from PDL were isolated by magnetic cell sorting and characterized by flow cytometry and immunostaining, as previously described.^15^ PDLMCs were grown in alpha Minimum Essential Medium (α-MEM) (Gibco, New York, USA) with 10% fetal bovine serum (FBS; Gibco, New York, USA) and 1% penicillin/streptomycin (P/S; FBS, Gibco, New York, USA) at 37°C in the atmosphere containing 5% CO_2_. These three PDLMCs populations were previously characterized as low osteoblast potential (LOP cells) according to quantification of mineral nodule deposition *in vitro* by Alizarin Red Staining (AR-S).^30^ They were frozen in Recovery Cell Culture Freezing Medium (Gibco, New York, USA) and kept in liquid nitrogen for subsequent experiments. For each experiment, cells at passages from 3–5 were used in triplicate. Experiments were performed in triplicate three times, with comparable results obtained for each one.

### Cell viability

Cell viability of LOP cells was determined using 3-(4,5-dimethylthiazol-2-yl)-2,5-diphenyl tetrazolium bromide (MTT) assay (Thermo Scientific, New York, USA) on days 1,3, 7 and 10. When cells were cultured to approximately 80% confluence, they were seeded in 96-well plates (0.5 x 10^4^cells/well) in standard medium (α-MEM supplemented with 2% FBS and 1% P/S) and incubated in a humidified incubator at 37°C and 5% CO_2_ for 24h to allow cell adhesion. After 24 hours, medium was changed according to the experimental groups: (1) CONTROL - cells cultured in standard medium, (2) OM - cells cultured in osteogenic medium composed by standard medium supplemented with 50 mg/mL ascorbic acid (Sigma-Aldrich, Burlington, USA), 10 mM b- glycerophosphate (Sigma-Aldrich, Burlington, USA), and 10^-5^ M dexamethasone (Sigma-Aldrich, Burlington, USA), (3) OM + VD - cells cultured in OM supplemented with 1nM of 1,25(OH)_2_D_3_ (vitamin D_3_; Sigma-Aldrich, Burlington, USA). Cells were cultured for 10 days, and the medium was changed every other day. Experiments were performed in triplicate three times, with comparable results obtained on each occasion (technical replicates) for absorbance reading at 570 nm using VersaMax microplate reader (Molecular Devices).

### Real-time PCR

For RNA isolation, LOP cells were seeded (20 × 10^4^ cells/well) in 60 cm^2^ plates with standard medium and incubated for 24 h. After this period, the medium was changed according to the experimental groups described above, and cells were cultured for three, seven, and 14 days. At every time point, cells were lyzed for total RNA extraction using TRIzol reagent (Invitrogen, San Diego, CA, USA), followed by a phenol/chloroform extraction, and isopropanol precipitation. RNA samples were treated with Turbo DNA-free to remove genomic DNA (Invitrogen, San Diego, CA, USA). Single-stranded complementary DNA (cDNA) was synthesized from 2 μg total DNA-free RNA using First-strand cDNA synthesis (Roche Applied

Science, New York, USA) following the manufacturer’s recommendations for a final volume of 20 μL.

The primers for 1*8S, RUNX2, ALP, OCN, BMP-2, ASPN*, and *VDR* (Table 1) were designed using Primer3web version 4.1.0^31^ and sequences confirmed by UCSC PCR *in silico*.^32^ For subsequent analysis of secondary structures and confirmation of annealing temperature, the Beacon Designer free edition was used (Premier Biosoft International). The efficiency of reactions for each “primer” was optimized prior to the start of the RT-qPCR reactions themselves. The real-time PCR reaction was performed with the LightCycler 480 system (Roche Diagnostics GmbH, USA) using the “LightCycler 480 SYBR Green I Master” kit (Roche Diagnostics GmbH). The reaction profile was determined by a formula suggested by the equipment manufacturer. For each run, water was used as a negative control, and the reaction product was quantified using the LightCycler Relative Quantification Software (Roche Diagnostics GmbH). β-actin, *GAPDH*, and *18S* genes were evaluated according to their expression stability by algorithms (NormFinder software – MOMA). *18S* was chosen as the reference gene (“housekeeping”) for the normalization of values.

**Table 1:** Primer sequences used for real-time quantitative PCR amplifications.

Gene	Primer (5`- 3`sequences)	Anneling temperature	Product Size
18S	F: CGGACAGGATTGACAGATTGATAGC	51ºC	171 bp
	R: TGCCAGAGTCTCGTTCGTTATCG		
*RUNX2*	F: CCGTCCATCCACTCTACCAC	55º C	139 bp
	R: ATGAAATGCTTGGGAACTGC		
*ALP*	F: CGGGCACCATGAAGGAAA	55º C	184 bp
	R: GGCCAGACCAAAGATAGAGTT		
*OCN*	F: AGCTCAATCCGGACTGT	55º C	150 bp
	R: GGAAGAGGAAAGAAGGGTGC		
*ASPN*	F: CCAACCGCGAGAAGATGA	60º C	223 bp
	R: CCAAGCAAGGTCTTCCAAAG		
*BMP-2*	F: CTTCCACCCCTCTTTCTTCC	58º C	221 bp
	R: GTCTCCCGGAAACACTTGAA		
*VDR*	F: TATCCACCCAGCCCATTCTC	59º C	206 bp
	R: TCCCTCAACATCAGTCAGCA		

*RUNX2*: runt-related transcription factor 2; *ALP*: alkaline phosphatase; *OCN*: osteocalcin; *ASPN*: asporin; *BMP-2*: bone morphogenetic protein 2. *VDR*: vitamin D receptor.

### ELISA

Cells were seeded (2 × 10^4^ cells/well) in six-well culture plates and incubated overnight in standard medium. Next, cells were cultured under conditions described in section 2.3. The antigens were prepared to a final concentration of 80μg/mL using Phosphate Buffered Saline (PBS). The plate was sealed and incubated overnight at 4. The wells were washed three times with 400 μL of 1% Tween 20 in PBS. The remaining protein binding sites in the coated wells were then blocked by adding 200 µL of blocking buffer, 5% non-fat dry milk /PBS, per well. After washing, polyclonal rabbit antibody anti-*BMP2* (clone ab14933, Abcam, Cambridge, UK), diluted 1:100 in PBS, was added to the wells and incubated for 2 h. After another washing step, peroxidase-conjugated anti-rabbit IgG (Vector Laboratories, Inc., Burlingame, USA) diluted 1:1000 in PBS was added and maintained for 1 h. After the end of the reaction with 50 μL of 2 N H_2_SO_4_, (stop solution) absorbance was read at 450 nm (Genesys TM 2, Thermo Spectronic US, Rochester, NY, USA). *BMP-2* levels, as represented by absorbance values, were calculated considering the 3-day control as 100%.

### Osteogenic differentiation

To evaluate the capacity of mineral matrix deposition, cells were seeded (3x10^4^ cells/well) in 24-well plates and incubated for 24 hours in standard medium. Then, the medium was replaced according to experimental groups, and cells were maintained under osteogenic condition for 28 days, with the osteogenic medium being changed every three days. On designated days up to day 28, the ability of cells to promote mineral nodule formation was determined by Alizarin Red Staining (AR-S).^33^ After the discoloration procedure of mineral nodules, quantitative analysis (562 nm) was performed using VersaMax microplate reader (Molecular Devices).

### Statistical analysis

All experiments were performed in triplicate and repeated on at least three separate occasions. Data were initially examined for normality by Shapiro-Wilk test and expressed as mean ± standard deviation (SD). The differences between multiple groups were analyzed using two-way analysis of variance (ANOVA), followed by Tukey’s post-hoc test. Pearson correlation was used to calculate crosstalk between mRNA levels of *ASPN* and *BMP-2* on day 14. A significance level of less than 0.05 was adopted (GraphPad Prism 10 – GraphPad Software Inc). The data are shown as intragroup analysis, in which they are compared for the effect of the treatments over time, in addition to evaluating the differences among the treatments in each time point. In this way, the intragroup variables refer to the different times that the treatments are being subjected to, and the intergroup analyses relate to the different types of treatment.

## Results

### Metabolic activity of LOP cells is not affected by 1,25(OH)2D3

**Figure 1:**
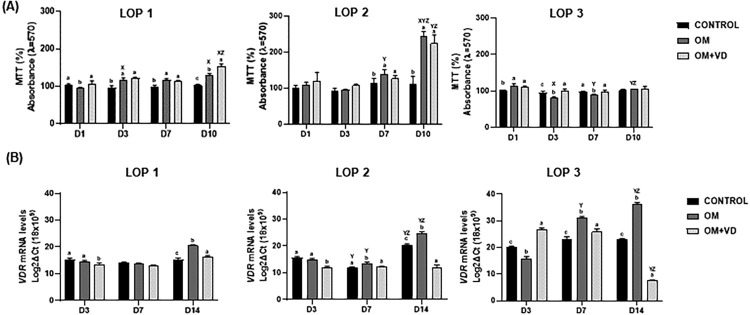
(A) Effect of 1,25(OH)2D3 on metabolic activity. Three LOP cell populations (LOP 1, LOP 2, and LOP 3) were cultured in standard medium (CONTROL), osteogenic medium (OM), and OM + 1nM of 1,25(OH)2D3 (OM + VD). MTT assay to assess cell metabolism and viability was performed at one, three, seven and 10 days. The control represents 100% of viability. (B) *VDR* expression after 1,25(OH)2D3 treatment. Real-time PCR analysis after treatment confirmed that all three populations express mRNAs for *VDR*. Experiments were performed in triplicate three times, with comparable results obtained on each occasion. Bars represent mean ± standard deviation (SD), intergroup analysis statistical differences are indicated by different lowercase letters, and intragroup statistically significant differences are indicated by different uppercase letters. The letter “X” represents the difference in relation to day one, while the letter “Y” represents the difference in relation to day three and “Z'” to day seven (P<0.05).

To verify whether LOP cells responded to 1,25(OH)_2_D_3_ treatment, metabolic activity was measured using MTT assay, as an indicator for cell viability. First, a range of concentrations of 1,25(OH)_2_D_3_ (0.1, 1, 10, 20 and 30 nM) was tested on a single population (LOP 1 cells). By the results, we verified that cell viability under 1,25(OH)_2_D_3_ treatment was dose-dependent (supporting data, Figure S1) with the concentration of 1nM not affecting the proliferation of LOP 1 cells during a 10-day period, when compared to CONTROL and OM groups. Then, all three LOP cell populations were treated with 1nM of 1,25(OH)_2_D_3_ to evaluate cell metabolic activity. The MTT assay results showed that the three LOP cell populations responded in different ways, but not unfavorably to 1,25(OH)_2_D_3_ treatment. LOP 1 cells have already shown an increase of metabolic activity after one day of 1,25(OH)_2_D_3_ treatment (absorbance (%)=106.6), which was significantly different when compared to OM group (absorbance (%)=94.3) (Figure 1) (P=0.007). This increase in metabolic activity was maintained throughout the 1,25(OH)_2_D_3_ treatment with highest levels after 10 days (Figure 1A) (P<0.0001). LOP 2 cells also had an improvement on day 10 of 1,25(OH)_2_D_3_ treatment (absorbance (%) = 223.25), but there was not a significant difference when compared to OM group (absorbance (%)=242.1) (P=0.16) (Figure 1). Finally, in LOP 3 cells, 1,25(OH)_2_D_3_ induced a significantly different increase of metabolic activity on day one (absorbance (%)=111.4), when compared to CONTROL group (absorbance (%)=101) (Figure 1) (P=0.0069). Further, after three and seven days, OM + VD group showed a higher metabolic activity (absorbance (%)=102; absorbance (%)=98.2, respectively) when compared to OM group, (absorbance (%)=82.17; absorbance (%)=90.32, respectively) (Figure 1) (P<0.0001; P=0.046 respectively). In summary, the results show that 1,25(OH)_2_D_3_ was not able to affect the viability of LOP cells.

### *VDR* expression

Quantitative qRT-PCR analysis confirmed that cell populations expressed the vitamin D receptor (*VDR*) gene. During the first three days, LOP 3 was the only one whose *VDR* expression was increased by 1,25(OH)_2_D_3_ (mRNA=26.70) (P<0.05), while LOP 1 and LOP 2 showed decreases in *VDR* expression in OM + VD (mRNA=13.55; mRNA=11.78 respectively) (Figure 1B). On day seven, LOP 2 and LOP 3 had increased *VDR* expression only in OM (Figure 1B) (P<0.05). It should be noted that the three cell populations showed a trend after 14 days of induction, that is, after this period in osteogenic medium (OM), the cells began to express considerably higher levels of *VDR* mRNA when compared to CONTROL and 1,25(OH)_2_D_3_ medium. In addition, during this same period it was clear that OM + VD reduced the expression of the receptor, even when compared to the CONTROL group (Figure 1B) (P<0.05).

### 1,25(OH)2D3 treatment changes the gene expression for *ASPN* and *BMP-2*

**Figure 2:**
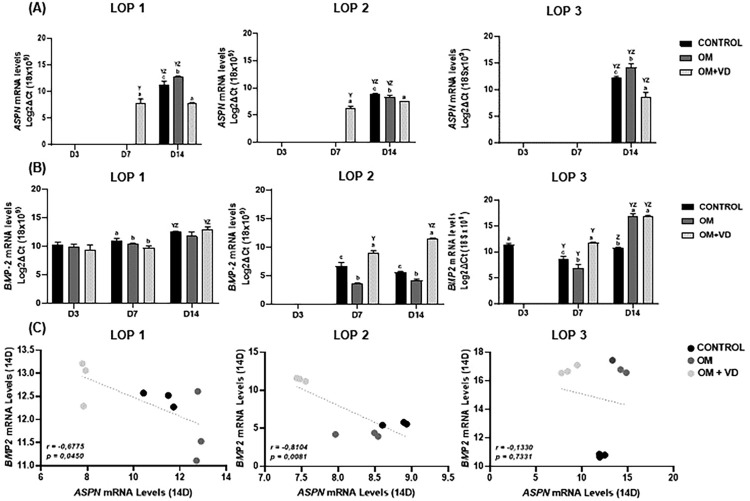
Real-time PCR analysis indicated a downregulation of the mRNA levels for *ASPN* (A), and an increased level of transcripts for *BMP-2* (B) after the treatment with 1nm of 1,25(OH)2D3. (C) Correlation between *BMP-2* and *ASPN* on day 14. Experiments were performed in triplicate three times, with comparable results obtained on each occasion. Bars represent mean ± standard deviation (SD), intergroup analysis statistical differences are indicated by different lowercase letters, and intragroup statistical significant differences are indicated by different uppercase letters. The letter “Y” represents the difference in relation to day three, while “Z” represents a difference in relation to day seven. (P<0.05)

LOP cells were cultured in standard medium (CONTROL), OM, and OM + 1 nM of 1,25(OH)_2_D_3_ for three, seven, and 14 days, and the effect on the gene expression for *ASPN* and *BMP-2* was detected using qRT-PCR. The results showed that in CONTROL and OM conditions, the *ASPN* gene is expressed by LOP cells only after 14 days (Figure 2A). Also, after 14 days of 1,25(OH)_2_D_3_ treatment (OM + VD) the mRNA levels for *ASPN* were significantly decreased compared with CONTROL and OM groups (Figure 2A) (P<0.05). At the same time, LOP 2 and LOP 3 cells had an increased expression of *BMP-2* gene from seven days of 1,25(OH)_2_D_3_ treatment (mRNA=9.02; mRNA=11.71 respectively), which was significantly different compared to the CONTROL and OM groups (Figure 2B) (P<0.05). In a different way, LOP 1 cells already expressed *BMP-2*, however, from three days in all cultured conditions (Figure 2B). Further, the mRNA levels for *BMP-2* increased from day three to day 14 (P<0.05), and no difference was observed in the OM+VD compared to CONTROL and OM groups (Figure 2B) (P>0.05). In summary, PCR results showed that 1nM of 1,25(OH)_2_D_3_ alters the pattern of *ASPN* expression in LOP cells after 14 days of treatment. However, the effect of 1,25(OH)_2_D_3_ on increasing *BMP-2* gene expression was favorable only for LOP 2 and LOP 3 cell populations.

Pearson correlation showed a negative correlation between *BMP-2* and *ASPN* in LOP 1 (r =-0.6775) and LOP 2 (r=-0.8104) over the 14-day period (Figure 2C). This shows a relation between a decrease in *ASPN* transcripts and an increase in *BMP-2* transcripts (P<0.05). In LOP 3, there was a trend towards a negative correlation between *ASPN* and *BMP-2*, which was not as statistically significant as in the other populations (Figure 2C) (r=-0.1330). In this sense, in LOP 3 the lower expression of *ASPN* did not result in a higher expression of *BMP-2* (p=0.7331).

### 1,25(OH)2D3 alters the expression for osteogenic gene markers

**Figure 3:**
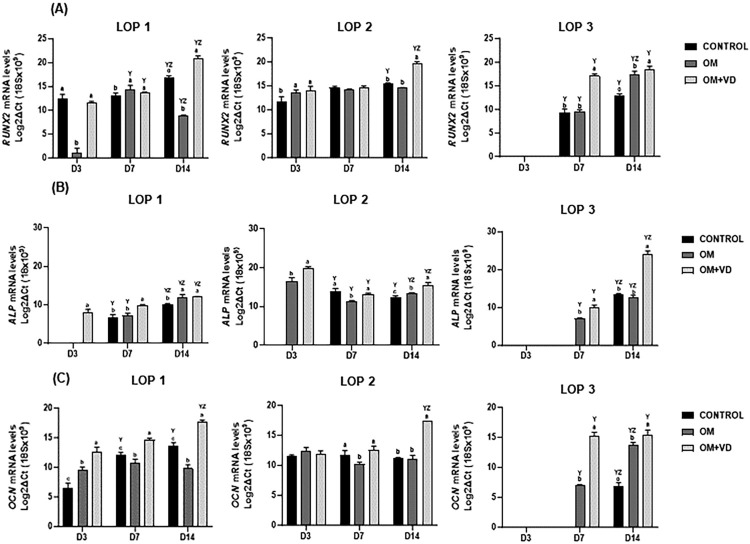
Osteoblast genes expression after 1,25(OH)2D3 treatment. Real-time PCR analysis after treatment indicated that 1,25(OH)2D3 upregulated the expression of mRNAs for *RUNX2* (A), *ALP* (B), and *OCN* (C). Bars represent mean ± standard deviation (SD), intergroup analysis statistical differences are indicated by different lowercase letters, and intragroup statistical significant differences are indicated by different uppercase letters. The letter “Y” represents the difference in relation to day three, while “Z” represents a difference in relation to day seven. (P<0.05).

To evaluate whether the modulation of expression for *ASPN* and *BMP-2* may enhance the osteogenic potential of LOP cells, mRNA levels for *RUNX2, ALP*, and *OCN* were detected by qRT-PCR. LOP 1, LOP 2, and LOP 3 cells showed an increase of expression for *RUNX2* gene after 14 days of 1,25(OH)_2_D_3_ treatment, when compared to CONTROL and OM groups (Figure 3A) (P<0.05). Both cells expressed significant levels of *ALP* transcripts after 3 days of treatment with 1,25(OH)_2_D_3_ (Figure 3B) (P<0.05). Concerning *OCN*, LOP 1 cells already showed a significant increase of mRNA levels on the third day of 1,25(OH)_2_D_3_ treatment (mRNA=12.6), while in LOP 2 cells this increase was only observed after 14 days (mRNA=17.31) (Figure 3B). In LOP 3 cells, the expression of genes for *RUNX2, ALP*, and *OCN* was only detected after seven days of culturing (Figure 3A-3C). The transcripts levels for *RUNX2* and *OCN* were significantly higher in the OM + VD group as early as day seven when compared to OM group (Figure3A and 3C) (P<0.0001). On the other hand, the expression of *ALP* in the OM + VD group was only significantly different from OM at day 14 (Figure 3B) (P=0.04).

### Expression of *BMP-2* protein is modified in three LOP cells by 1,25(OH)2D3

**Figure 4:**
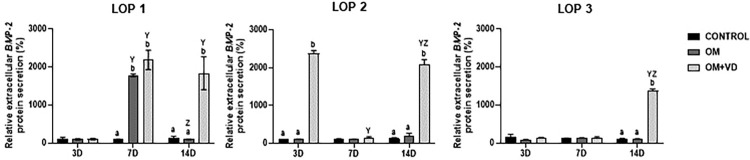
*BMP-2* protein expression. Quantification of *BMP-2* secreted in supernatant of LOP 1, LOP 2, and LOP 3 cells after three, seven, and 14 days of osteogenic induction associated with 1,25(OH)2D3. Bars represent mean ± standard deviation (SD), intergroup analysis statistical differences are indicated by different lowercase letters, and intragroup statistical significantly differences are indicated by different uppercase letters. The letter “Y” represents the difference in relation to day three, while “Z” represents a difference in relation to day seven. (P<0.05)

The ELISA assay results showed significantly higher levels of *BMP-2* in supernatants of LOP 1, LOP 2, and LOP 3 cells after 1,25(OH)_2_D_3_ induction compared to OM and CONTROL groups (Figure 4) (P<0.05). Although the *BMP-2* protein was more highly expressed in the 1,25(OH)_2_D_3_-induced groups, each cell population showed its own particularities regarding time. In LOP 1, there was a peak in *BMP-2* expression in the 1,25(OH)_2_D_3_ groups at seven and 14 days (absorbance (%)=2188.85; absorbance (%)=1839.68 respectively). In LOP 2, the peaks occurred on days three (absorbance (%)=2375.4) and 14 (absorbance (%)=2093.43), while in LOP 3 only on day 14 (1375.29) (Figure 4). (P<0.05)

### 1,25(OH)2D3 increases mineralized matrix deposition by LOP cells

**Figure 5:**
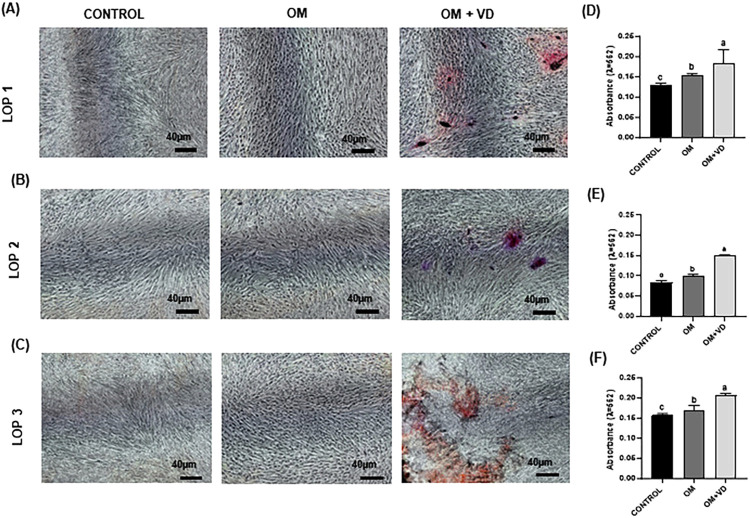
Effect of 1,25(OH)2D3 on mineral nodule deposition. (A-C) Microscopic of Alizarin Red Staining (AR-S) mineralization of LOP 1, LOP 2, and LOP 3, respectively. Scale bar = 40µm. (D-F) Quantification of AR-S at 28 days. Experiments were performed in triplicate three times, with comparable results obtained on each occasion. Bars represent mean ± standard deviation (SD) and intergroup analysis statistical differences are indicated by different lowercase letters (P<0.05).

To verify the long-term effect of 1,25(OH)_2_D_3_ on osteoblast differentiation, AR-S assay was used to identify mineralized nodule deposition *in vitro*. After 28 days of culturing, AR-S revealed that the osteogenic medium did not induce a significant mineral nodule deposition (Figure 5A-5C). On the other hand, OM + VD treatment increased mineralization compared to osteogenic medium alone in all LOP cell populations (Figure 5A-5F). (P<0.05)

## Discussion

This study was conducted to confirm the osteogenic effect of 1,25(OH)_2_D_3_ in three LOP cells of PDLMSCs. After 14 days of 1,25(OH)_2_D_3_ treatment, the mRNA levels for *ASPN* and *VDR* decreased (P<0.05), while *BMP-2* transcripts and extracellular expression increased (P<0.05). In parallel, *RUNX2, ALP*, and *OCN* gene expression was upregulated by 1,25(OH)_2_D_3_ treatment, resulting in an increase of mineral nodule deposition *in vitro* (P<0.05). Contradicting the null hypothesis, 1,25(OH)_2_D_3_ obtained favorable results in terms of mineralized matrix production when compared to the OM group. In this sense, to the best of our knowledge, it is the first time that 1,25(OH)_2_D_3_ has been shown to alter the osteoblastic capacity of cells with low osteoblast potential.

There is a pressing need to find therapies with broad indications and greater predictability for periodontal regeneration. Recent research has sought to analyze ways of enhancing the osteo-cementoblastic differentiation capacity of PDLMCs,^9-12^ which are quite heterogeneous in character, and few have demonstrated the ability to produce mineralized matrix *in vitro*.^14-16^ Studies have shown that *ASPN* is a negative regulator in the differentiation of mesenchymal cells of the periodontal ligament.^18^ At the same time, *ASPN* is known to competitively bind to *BMP-2*, resulting in inhibition of *BMP-2* activation.^18,34^ It was also related that 1,25(OH)_2_D_3_ had an inhibitory effect on *ASPN* transcription.^29^

The maintenance of appropriate vitamin D levels has shown to be associated with better oral development and health throughout life.^35^ Although the influence of Vitamin D_3_ on the mesenchymal cells of the periodontal ligament is still not entirely clear, it was reported that vitamin D_3_ promotes osteogenic differentiation of human PDL cells and antagonizes inflammation in human periodontal tissue.^36^ Knowing that vitamin D_3_ can act as a negative regulator of *ASPN*, our study is the first to determine its effect in three different PDL-CD105^+^ cell populations characterized as low osteoblast potential.

According to the MTT results obtained, 1nM of 1,25(OH)2D_3_ did not compromise the metabolic capacity of all the populations. Moreover, we found that after 10 days of induction, OM + VD had the ability to be neutral or improve cellular viability. Vitamin D_3_ can reduce the expression of pro-inflammatory cytokines, suggesting promotion of periodontal regeneration and antagonizing periodontal inflammation.^29,36^ Vitamin D_3_ also plays a significant role in various aspects of cell functions and is associated with the regulation of osteoblastic and osteoclastic activities, thereby impacting both the resorption and synthesis stages of bone remodeling.^37^

It has been suggested that cells of the periodontal ligament can metabolize Vitamin D due to the presence of receptors.^24,25^ *VDR* mediates the transcriptional actions of the hormonally active form of vitamin D3 (1,25(OH)_2_D_3_).^38^ Our qRT-PCR analysis confirmed that all cell populations expressed the vitamin D receptor gene. We noticed a pattern on the fourteenth day in which LOP 1, LOP 2, and LOP 3 had decreased *VDR* transcripts in the presence of Vitamin D. 1,25(OH)2D_3_ autoregulates the expression of the *VDR* gene in response to exposure to Vitamin D_3._^38^ Therefore, we can suggest that the decrease in *VDR* expression is related to presence of the active metabolite there, no longer requiring the receptor metabolizing function. On the other hand, the OM group considerably increased *VDR* expression levels. Dexamethasone, one of the components of osteogenic induction medium, has been reported to up-regulate mRNA expression of *VDR* in primary cultures of osteoblast-like cells.^39^

Considering the primary cell culture and its heterogeneity, all of them showed reduced *ASPN* expression after 14 days of OM+VD induction. On the other hand, *BMP-2* had its mRNA expression improved by 1,25(OH)_2_D_3_ after seven days of induction in most populations. Thus, we can observe that *ASPN* started to be limited by 1,25(OH)_2_D_3_ only on the 14^th^ day, which may suggest that, between seven and 14 days, the signaling pathway started to receive the influence of *BMP-2* and had the expected reduction in the last period. Zhang, et al.^29^ (2020) confirmed that a vitamin D receptor (*VDR*) binding site was identified in the *ASPN* promoter region, which can explain the negative regulation of vitamin D on *ASPN* gene expression.

After 14 days, cell populations LOP 1 and LOP 2 showed a negative correlation when comparing *BMP-2* and *ASPN*. That is, while *ASPN* expression was being reduced in the OM + VD group, *BMP-2* transcripts increased, confirming the influence between them.^18,34^ Although there was a negative correlation trend for LOP 3, it was not possible to state that *ASPN* was influencing *BMP-2* expression. This does not mean that 1,25(OH)2D_3_ had no effect, but its action extent could not interfere with *BMP-2* expression, which remained similar to the OM group.

To confirm the stimulatory effect of 1,25(OH)_2_D_3_ on *BMP-2* protein expression, we noticed that all cell populations began to express more extracellular protein in the Vitamin D groups, especially after 14 days. The difference with the CONTROL and OM groups was evident, corroborating the gene expression data, especially in LOP 1 and LOP 2, which confirmed the correlation with *ASPN*. Although the OM + VD groups from LOP 1 and LOP 2 showed high protein expression on days seven and three respectively, there was a unanimous increase in extracellular *BMP-2* protein after 14 days. Therefore, we suggest that 1,25(OH)_2_D_3_ induced cells to secrete more *BMP-2* proteins, which may have contributed to the mediation and alteration of the low osteoblastic potential of LOP 1, LOP 2, and LOP 3. It is important to note that *BMP-2* is a key factor in this phenotype change, since this protein signaling regulates bone mineral density and it is a growth factor that initiates osteoblast differentiation.^40^

The microscopic analysis of Alizarin Red Staining (AR-S) after 28 days of induction showed more evident mineral nodules in the OM + VD group. These data were confirmed by ARS quantification . The greater formation of mineral nodules may suggest that the phenotype of these three cell populations with low osteoblast potential could be altered by the action of 1,25(OH)_2_D_3_. The AR-S was essential to highlight mineralization and the difference between the groups. Vitamin D_3_ has been demonstrated to regulate osteoblast and chondrocyte gene transcription, proliferation, and differentiation.^37^

It has been reported that 1,25(OH)2D_3_ enhanced the expression of runt-related transcription factor 2 (*RUNX2*), osteocalcin (*OCN*), and alkaline phosphatase (*ALP*) in human osteoblastic cells.^36,41^ Confirming our hypothesis, the highest transcript expressions of *ALP, RUNX2, and OCN* occurred in the presence of 1,25(OH)_2_D_3_. *ALP* activity is commonly assessed as an early marker of osteogenic differentiation,^42^ as it plays a crucial role in bone mineralization by initiating and/or promoting hydroxyapatite crystal formation in the osteoblast matrix.^43^ On the other hand, *RUNX2* has been identified as a “master gene” in controlling osteogenic differentiation,^44^ serving as an early-stage transcription factor that activates osteoblastic differentiation.^45,46^ It triggers *OCN* transcription by binding to target promoters and enhancers.^47^ *RUNX2* expression regulates bone matrix mineralization, and its upregulation is associated with enhanced stimulation of the extracellular matrix mineralization.^48,49^ The *OCN* gene is a marker for late-stage osteogenic differentiation and is synthesized only during the maturation of osteoblasts, odontoblasts, and cementoblasts.^44,50^ Therefore, the greater expression of these osteogenic markers in the OM + VD group helps to confirm the potential of 1,25(OH)2D_3_ to stimulate O/C differentiation.

However, it must be emphasized that more tests are needed to understand the modulation between *ASPN* and *BMP-2* and its consequence for the osteogenic differentiation of PDLMSCs. Also, *in vitro* study conditions can oversimplify complex interactions, leading to results that might not translate directly to *in vivo* settings, which can limit the applicability. Furthermore, new studies are needed to visualize the broad influence that 1,25(OH)_2_D_3_ can have on periodontal ligament cells *in vivo*, to establish the possibility of local application as a future alternative for periodontal regeneration.

## Conclusion

The study demonstrates that treatment with 1,25(OH)_2_D_3_ enhances the differentiation potential of PDL-CD105+ cells with initially low osteoblast/cementoblast potential. This enhancement is evidenced by an increased production of mineralized matrix and accompanied by alterations in the gene expression of *ASPN* and *BMP-2*. These findings suggest that 1,25(OH)_2_D_3_ effectively improves the osteogenic profile of PDL cells that initially showed low predisposition for mineralized tissue reconstruction. Future steps will involve *in vivo* treatments to further evaluate the clinical effects of 1,25(OH)_2_D_3_.
